# Potential revenue from taxing e-cigarettes and comparison of annual costs of daily e-cigarette use versus daily cigarette smoking among South African adults

**DOI:** 10.18332/tid/131861

**Published:** 2021-01-29

**Authors:** Israel T. Agaku, Catherine O. Egbe, Olalekan A. Ayo-Yusuf

**Affiliations:** 1 School of Health Systems and Public Health, University of Pretoria, Pretoria, South Africa; 2 Department of Oral Health Policy and Epidemiology, Harvard School of Dental Medicine, Boston, United States; 3 Alcohol, Tobacco and Other Drug Research Unit, South African Medical Research Council, Pretoria, South Africa; 4 Department of Public Health, Sefako Makgatho Health Sciences University, Pretoria, South Africa; 5 Africa Centre for Tobacco Industry Monitoring and Policy Research, Sefako Makgatho Health Sciences University, Pretoria, South Africa

**Keywords:** cost, taxation, e-cigarettes, sales

## Abstract

**INTRODUCTION:**

To inform policy making under the proposed *The Control of Tobacco and Electronic Delivery Systems Bill*, we compared annual costs of using e-cigarettes versus cigarettes, and estimated revenue from e-cigarette taxation.

**METHODS:**

We extracted e-cigarette retail prices from 231 South African e-cigarette vendor websites. We compared annual costs associated with daily cigarette smoking (self-reports from daily smokers in the 2018 South African Social Attitudes Survey, SASAS) versus daily e-cigarette use (based on cumulative costs of consumables plus device costs). We estimated revenue from excise tax if e-cigarettes were taxed at 75% (the rate proposed by the government) and 37.5% (half of the government’s proposal as a hypothetical scenario) of the cigarette excise rate. We applied the different rates to e-cigarette consumption in 2018 SASAS and projected for 2021.

**RESULTS:**

Mean annual cost associated with daily use was ZAR 6693 (US$460.32, based on an exchange rate of about 69 US$ to 1000 ZAR) for manufactured cigarettes; for e-cigarettes, this ranged from ZAR 8574.69/year (with price minimizing strategies) to ZAR 19780.83/year (retail products exclusively). Expected revenue from e-cigarette excise tax at 75% of the cigarette tax rate was up to ZAR 2.20 billion (95% CI: 0.96–3.44). If taxed at 37.5% of the cigarette tax rate – half of the government’s proposed rate – the projected revenue was up to ZAR 1.10 billion (95% CI: 0.48–1.72). Of the projected revenue from e-cigarette excise tax at 75% of the cigarette rate, the portion attributable to hardware (device and batteries) was 61% (ZAR 1.35 billion), while the portion attributable to e-liquid was 39% (ZAR 0.86 billion).

**CONCLUSIONS:**

Calculated daily costs were higher for e-cigarettes than cigarettes. We recommend an e-cigarette excise tax. The government’s proposed tax rate may reduce youth e-cigarette access, while allowing adult smokers wishing to switch exclusively to e-cigarettes to reduce their tobacco-related harm.

## INTRODUCTION

Since their introduction to the South African market a decade ago, e-cigarette sales have increased while cigarette sales declined^1^. E-cigarettes are not regulated under the Tobacco Products Control Act of 1993^2-4^. Although e-cigarettes are required to be registered with the Medicines Control Council for legal sale; they are currently promoted as consumer products^5^.

To protect youth from e-cigarettes, South Africa proposed *The Control of Tobacco and Electronic Delivery Systems Bill*, which aims to regulate e-cigarettes as traditional tobacco products^6^. To inform policy making on e-cigarettes in South Africa, data are needed on price, especially as this is a major determinant of demand among youth^7^. Data on pricing are especially important to evaluate the claim by the e-cigarette industry that e-cigarettes are cheaper than traditional cigarettes^8^, a claim that seeks to increase product appeal, acceptance, and use. Both cigarette smokers and e-cigarette users are known to utilize various price-minimizing strategies, and estimation of price must account for these various tax avoidance strategies^9,10^.

This study has two objectives. The first is to assess comparative costs of using e-cigarettes versus cigarettes daily among South African adults. Analyzing daily users ensured balanced comparisons using reasonably ‘exchangeable’ groups. Second, we estimated how much revenue can be generated from implementing excise taxes on e-cigarettes in South Africa.

## METHODS

### Data sources and measures

The parameters used to answer the key study questions were sourced from different datasets. Supplementary file Table S1 describes the various data sources used in this study. The study was approved by the University of Pretoria’s Faculty of Health Sciences’ Ethics Review (No. 39/2019).

#### Product characteristics and marketing strategies for e-cigarettes

From a comprehensive list of South African e-cigarette retail websites, which we created in June–July 2020 (n=231 unique websites), we extracted information on brand, volume (mL), concentration (mg), and price, for 2661 refill liquids and 171 hardware. There was a two-step process in accessing e-cigarette products sold by online vendors. First, using Google Place and social media sites like Facebook, Twitter, and South African online e-cigarette forums, we identified existing vape shops in South Africa (both brick-and-mortar, and online). The websites for the vape shops were also obtained. Next, using web scrapping in Python, we extracted information on marketed products for all distinct vendor websites.

#### Comparative costs of daily smoking of manufactured cigarettes versus daily e-cigarette use

To estimate costs of daily cigarettes among a nationally representative sample of South African adults aged ≥16 years, we used data from the 2018 South African Social Attitudes Survey (SASAS, n=2736). Daily smokers of ‘manufactured cigarettes’ reported smoking ‘currently every day’ when asked: ‘Do you use or have you used any of the following tobacco products? – manufactured cigarettes’. Cigarettes smoked per day were assessed as follows: ‘On the days that you smoke, on average, how many manufactured cigarettes (excluding hand rolled cigarettes) do you smoke per day? If less than one a day, type 0’. We replaced responses of ‘0’ with 0.5 conservatively (7 of 384 daily smokers), otherwise, monthly cigarette counts for those daily smokers would erroneously yield zero. Associated costs from the last purchase made was assessed with the questions: 1) ‘How much did you pay for your last cigarette purchase, per stick/individual cigarette?’ (for those who bought single sticks); and 2) ‘How much did you pay for your last cigarette purchase, per pack?’ (for those who bought a pack).

We examined both perceived and actual e-cigarettes costs. For perceived affordability, we explored data from a web-based survey of South African adults aged ≥18 years conducted during 25 June to 9 July 2018 (Health 24 survey, n=18208). Participants in this survey were asked: ‘To what extent do you agree or disagree with each of these statements about smokeless products – e-cigarettes, vaping and heat-not-burn devices are too expensive to use?’. Those answering ‘agree/strongly agree’ were classified as perceiving these products as being ‘too expensive’; responses of ‘strongly disagree’, ‘disagree’, and ‘neutral’ were classified as absence of an affirmative response. To estimate actual costs of daily e-cigarette use, assumptions were: 1) being habitual, e-cigarette use was likely with a ‘mod’ device to better customize vaping sensory experience, and 2) only one mod was purchased yearly. The average retail costs of devices, refill liquids and other consumables (e.g. coil, batteries, cotton wool) were obtained from the online vendor data whereas typical consumption patterns/frequencies were from an online forum of e-cigarette users in South Africa known as ‘Ecig Vape Forum South Africa’ (ECIGSSA); this is South Africa’s largest e-cigarette forum, with over 8000 members^10^. One of the authors, IA, registered on the forum on 9 April 2019 and posted the following question: ‘Curious how much variation in total MONTHLY cost from vaping is within the vaping community and how this differs between newbies versus the more experienced vapers. Please, can you tell me your average monthly cost and how long you have been vaping?’. A total of 20 responses to the question were analyzed (Supplementary file Table S2). Users also provided estimates of the amount of e-cigarette liquid they consumed weekly; the modal response was approximately 200 mL/week (e.g. ‘about 60 mL of juice every 2nd day’ or ‘pretty much 2 × 100 mL a week’). We conservatively used half of this quantity (100 mL/week) as representing the ‘average’ user, given the possibility that users on dedicated e-cigarette forums such as ECIGSSA may be outliers in their pattern of e-cigarette use. For e-cigarette price minimizing strategies, we estimated costs assuming consumables like coils or refill liquids were homemade (i.e. do-it-yourself or DIY).

#### Potential revenue from taxation of e-cigarettes

Our framework for estimating e-cigarette taxes is underpinned by the following philosophical principles:

(1) When used exclusively in lieu of cigarettes, e-cigarettes have a relatively lower harm profile compared to cigarettes as acknowledged by the National Academy of Sciences consensus report on e-cigarettes: ‘There is conclusive evidence that completely substituting e-cigarettes for combustible tobacco cigarettes reduces users' exposure to numerous toxicants and carcinogens present in combustible tobacco cigarettes’^11^. Reflecting this, the e-cigarette tax should be lower than cigarettes. The South African Finance Minister announced plans for excise tax on heated tobacco products at the rate of 75% of the cigarette tax rate in cognizance of the potential for harm reduction^12,13^. In this study, we estimate potential revenues from implementing e-cigarette excise tax at the announced rate of 75% of the cigarette excise tax rate; we also made estimates at 37.5% of the cigarette excise tax rate (half of the proposed threshold) as a hypothetical worst case scenario to examine what the government revenue would be under this assumption (e.g., if we assumed the own price elasticity of demand for e-cigarettes was twice that for cigarettes).

(2) While e-cigarettes have some potential to help adult, non-pregnant smokers quit if used exclusively in lieu of cigarettes^14^, concerns exist regarding population-level harms such as youth initiation. Given that youth are generally price-sensitive, imposing excise taxes on e-cigarettes may discourage youth initiation while allowing adults to benefit from them. On the other hand, not taxing e-cigarettes would make them more affordable to youth and lead to an epidemic of use.

(3) To adopt a pragmatic approach that acknowledges real-world patterns of use and ensure effective tax collection, we focus on e-cigarette device, batteries (including replacements), and refill liquids, but not other consumables such as coils, which could easily be homemade. Similarly, our analyses assume that e-liquid used was mixed by the user from marketed concentrates as a price-minimization strategy (discussed below).

(4) Taxation of e-cigarettes should be based on ‘typical’ consumption patterns, bearing in mind that the ‘average’ South African e-cigarette user is behaviorally different from the ‘average’ South African cigarette smoker. Most current cigarette smokers are daily smokers, whereas most current e-cigarette users are only occasional users; in the 2018 SASAS, 65.3% and 75.5% of all ever and current smokers of manufactured cigarettes, respectively, were current daily smokers, whereas only 18.0% and 27.1% of ever and current e-cigarette users, respectively, used e-cigarettes every day. These considerations become relevant when deciding what amount of e-liquid consumption is equivalent to a cigarette pack for taxation purposes. We propose that 1 cigarette pack is equivalent to 100 mL of e-liquid based on ‘typical’ weekly cigarette consumption among non-daily cigarette smokers, and e-liquid consumption for the ‘typical’ e-cigarette user, who is a non-daily user as well. From 2018 SASAS, non-daily cigarette smokers reported 3 sticks as their median number of cigarettes smoked per day on the days they smoked (mean = 7.34). A pack of 20 cigarettes is therefore the average maximum quantity smoked per week for the non-daily cigarette smoker (considering the median cigarettes smoked per day) and could therefore be considered, for taxation purposes, equivalent to 100 mL of e-liquid, the average weekly consumption for e-cigarette users. Henceforth, we refer to 100 mL of ready-to-use e-liquid as 1 cigarette-pack-equivalent (CPE) of e-liquid for tax purposes, regardless of whether user-mixed or pre-mixed.

For our calculations, estimates are generated for 31 December of a given year and apply to the whole year. To estimate potential revenue from taxation of e-cigarettes, we examined hardware (device and batteries) separately from e-liquids. We assumed only one device per year, one replacement battery per month of usage, and e-liquid consumption based on *ad libitum* usage. We assumed e-cigarette users will mix their own liquid as a tax avoidance strategy (i.e. will DIY 100 mL of user-mixed liquid from 1 pack of 10 mL concentrate, rather than purchasing 100 mL of pre-mixed liquid). Device price used in our estimation was averaged across ‘mods’.

We divided e-cigarette users into four groups, by frequency of e-cigarette consumption, based on data in 2018 SASAS: 1) ‘every day’, 2) ‘some days’ 3) ‘stopped completely less than 6 months ago’, and 4) ‘stopped completely more than 6 months ago’. Prevalence and extrapolated counts were generated for each of these categories using nationally representative weights. Assumed consumption patterns, based on parameters obtained from ECIGSSA, were as follows:

Group 1 (daily users) – consume 1 CPE of e-liquid (100 mL) per week, 52 weeks in a year. From a DIY perspective, this is equivalent to 1 pack of 10 mL concentrate per week, or 52 packs of 10 mL concentrates per year, since each pack of 10 mL concentrate yields about 100 mL of vape juice on mixing.

Group 2 (non-daily users) – consume 75% of the e-liquid as the daily users since they also use e-cigarettes throughout the year, albeit non-daily.

Group 3 (regular users for half of the year) – consume 50% of e-liquid as the daily users as we assume, they used an e-cigarette at least all through the first half of the year.

Group 4 included not only individuals who may have used e-cigarettes for part of the current year before quitting, but also those who quit before the current year.

To estimate the number in just the current calendar year, we used an ‘all or none assumption’, i.e. that all of those individuals used e-cigarettes for parts of the current calendar or none of them used for any part of the year. A third assumption was that the number who quit in the earlier half of the current calendar year (i.e. subset of Group 4 restricted to the current year) was numerically equal to those who quit in the latter half of the year (i.e. Group 3). In analyzing Group 4, we applied 25% of the consumption rates of daily users to this group, assuming they used e-cigarettes for ≤3 months in the current calendar year.

### Analyses

All costs are presented in South African Rand (ZAR) (US$ 1 about ZAR 14.54). Annual costs were generated to compare costs associated with cigarette versus e-cigarette use. The daily cost of smoking cigarettes was the cost of a single pack × mean number of cigarettes smoked per day/20, where 20 represents the number of cigarette sticks in a standard pack. Monthly costs were extrapolated by multiplying daily costs by 30; annual costs by multiplying monthly costs by 12.

Weighted percentages were calculated to describe perceived costs of e-cigarette use from the online web survey of 18208 South African adults, stratified by various demographic, socioeconomic, and tobacco use characteristics. To estimate actual costs associated with pre-mixed e-liquid and other consumables purchased from retailers, we used the conservative estimate of typical weekly e-liquid consumption (100 mL/week). We multiplied the cost of a 100 mL pre-mixed e-liquid pack by 52 to generate the annual cost associated with using pre-mixed e-liquid. Cost of replacing other consumables, such as coils, was obtained by applying reported frequency of replacement from ECIGSSA, to the retail cost of the respective products.

Estimates associated with price-minimizing strategies for e-cigarette use were similarly calculated; we assumed user-mixed e-liquid rather than pre-mixed. From our online vendor data, a pack of 10 mL concentrate (which yields 100 mL of vape juice on mixing) costs ZAR 83.51, on average. We multiplied the cost of one pack of 10 mL concentrate by 52 to get the annual cost associated with user-mixed e-liquids. Costs associated with e-liquids were added to costs associated with other consumables (e.g. cotton, coils), as well as hardware costs to get the total annual cost of using e-cigarettes.

In South Africa, the Treasury targets approximately 40% excise tax on cigarettes (exclusive of value added tax, VAT, which is about 15%). To estimate the potential revenue from e-cigarette excise taxes in 2018 (year with available data), we applied the *a priori* specified e-cigarette tax rate of 30% (i.e. 75% of the cigarette excise tax, or 0.75×40), the 75% being the e-cigarette rate proposed by the South African Minister of Finance. We compared this to the hypothetical e-cigarette tax rate of 15% (i.e. 37.5% of the cigarette excise tax rate, or 0.375×40) – the 37.5% being half of the government’s proposed rate. For e-cigarette hardware (devices and batteries) where the price was known and relatively fixed, we applied the above excise taxes (30% and 15%). For e-liquids where the prices were highly variable depending on composition, we simply added a fixed nominal excise tax ZAR value on a cigarette-pack equivalent of e-cigarette liquid, calculated as 75% or 37.5% of the corresponding nominal value of the excise on a pack of regular cigarettes, which was ZAR 15.52/pack during 2018^15^. Hence, 75% of this amount yields ZAR 11.64, while 37.5% yields ZAR 5.82 per cigarette-pack-equivalent of e-cigarette liquid. Total tax was the sum of e-liquid and hardware taxes. We generated total projected taxes for 2021 from the 2018 estimates based on market forecasts for e-cigarettes in South Africa during 2018–2021 by Euromonitor International, a global market research firm (forecast: 24.9% increase in consumption during 2018–2021)^1^. Regression analyses were performed to explore the relationship between cost and product type using the online vendor data. Data were analyzed with Stata Version 14 in 2020.

### Sensitivity analyses

There is no benchmark to directly assess the validity of our projected tax revenue from e-cigarette excise taxes; we therefore assessed this indirectly by calculating expected cigarette excise tax with the same methodology as e-cigarettes above and comparing the generated estimate to known benchmarks for cigarettes (ZAR 12.5 billion in cigarette excise taxes in the 12-month period between April 2018 and March 2019)^15^. To estimate total cigarette excise taxes for 2018, we used the 2018 SASAS data. All parameters were self-reported. In brief, we summed expected annual consumption for daily and non-daily smokers. Total revenue on cigarettes by daily smokers assuming they smoked all 365 days of a year was:

Total cigarette expenditure =(Mean number of cigarettes per day × days smoked in a year/20) × average pack price

We assumed daily smokers smoked all 365 days in a year and that non-daily smokers smoked 75% of the year. Analyses also accounted for former smokers who smoked for part of the year as described above for e-cigarettes. We applied the cigarette excise tax rate of 40% to total cigarette expenditure during the one-year period exclusive of value added tax (i.e. calculations based on pre-VAT price) and generated the final expected revenue by subtracting lost revenue because of illicit cigarettes from the total projected excise tax. The extent of illicit trade was estimated in 2018 SASAS with the question: ‘Overall, how many of the cigarettes that you have smoked could possibly be counterfeit or illegal (tax not paid/smuggled)?’. Response options were ‘none’, ‘a little’, ‘about half’, ‘most’, and ‘all’. Using these responses, we estimated the magnitude of illicit trade as 33.21%, based on the sum of the following percentages with corresponding weights: % ‘none’ × 0, % ‘a little’ × 0.25, % ‘about half’ × 0.5, % ‘most’ × 0.75, and % ‘all’ × 1.0)^16^. Similar estimates of up to 30–35% for illicit cigarette trade in South Africa have been reported elsewhere^17^.

## RESULTS

Weighted counts from the 2018 SASAS showed that an estimated 1.09 million South African adults aged ≥16 years used e-cigarettes every day or some days during 2018 (295081 adults every day, or 0.73% prevalence; 794936 adults some days, or 1.98% prevalence). Furthermore, 0.8% of the entire adult population indicated they stopped e-cigarette use <6 months ago (320003 adults) while 0.58% (231369 adults) reported they stopped e-cigarette use >6 months ago. For manufactured cigarettes, 8.2 million individuals reported smoking every day or somedays (6196978 adults every day, or 15.42% prevalence; 2013620 adults some days, or 5.01% prevalence). Furthermore, 0.72% of the entire adult population indicated they stopped cigarette smoking <6 months ago (290850 adults) while 2.47% (991440 adults) stopped smoking >6 months ago.

### Characteristics of marketed e-cigarettes in South Africa

Cannabidiol (CBD) e-liquids were commonly available in one of the following three package sizes: 30 mL (32.7%, 251/767), 60 mL (33.5%, 257/767), and 120 mL (32.3%, 248/767). After adjusting for concentration, the unit price for CBD refill liquid increased by ZAR 2.1 with every 1 mL increase in package size (β=2.10; 95% CI: 1.98–2.22, p<0.001). A significant, but smaller increase in price was seen with every 1 mg increase in the concentration of CBD (β=0.93; 95% CI: 0.90–0.95, p<0.001). For nicotine-containing regular e-cigarette refill liquid, the package size, but not the nicotine concentration was associated with price. With every 1 mL increase in package size, the price increased by ZAR 0.67 (95% CI: 0.55–0.80, p<0.001). Mean prices for the different liquid types and package sizes are shown in Table 1.

**Table 1:** Characteristics and pricing of e-cigarette liquids, South Africa, 2020

*Product and available package sizes (mL)*	*Number (n)*	*Distribution (%)*	*Concentration (mg)*	*Sale price (ZAR)*
**Cannabidiol (CBD)**				
10	4	0.52	200.00	224.33
30	251	32.72	318.73	420.00
60	257	33.51	310.30	485.69
75	7	0.91	174.57	308.57
120	248	32.33	324.73	613.92
Total	767	100		
**Nicotine salts**				
1.2	4	1.45	60.00	110.00
15	2	0.72	20.00	100.00
30	241	87.32	28.85	214.45
60	28	10.14	11.71	258.18
100	1	0.36	20.00	210.00
Total	276	100		
**E-liquid concentrates**				
10	193	51.33	4.23	83.51
15	31	8.24	0.00	85.77
20	120	31.91	0.38	66.80
30	30	7.98	1.27	137.87
50	1	0.27	6.50	225.00
60	1	0.27	0.00	149.00
Total	376	100		
**Nicotine-free regular e-cigarette liquid**				
30	1	0.93	0.00	75.00
60	79	73.83	0.00	212.27
75	18	16.82	0.00	217.78
90	1	0.93	0.00	315.00
100	7	6.54	0.00	277.14
120	1	0.93	0.00	280.00
Total	107	100		
**Nicotine-containing regular e-cigarette liquid**				
30	141	12.42	3.63	220.78
50	5	0.44	3.00	293.00
60	575	50.66	3.49	224.80
65	23	2.03	3.00	258.22
70	3	0.26	3.00	250.00
75	37	3.26	3.16	231.16
80	5	0.44	3.00	212.00
90	2	0.18	4.50	315.00
100	154	13.57	2.92	274.44
120	190	16.74	3.22	268.68
Total	1135	100		

For hardware/device prices, we estimated the following means: disposable e-cigarettes ZAR 97.5, replacement coils ZAR 78.24, pod or pod-mod systems ZAR 604.42, mod systems ZAR 812.19, digital charger ZAR 356.67, and battery ZAR 210.

### Comparative costs of using regular versus electronic cigarettes

Average pack price from 2018 SASAS was ZAR 30.94, mean cigarettes per day were 12.02 for daily smokers and 7.34 for non-daily smokers on the days they smoked. Mean monthly cost associated with daily cigarette smoking was ZAR 558 and annual extrapolation was ZAR 6693.

Estimated annual cost of daily e-cigarette use ranged from ZAR 8574.69 with DIYs to ZAR 19780.83 without DIYs (Table 2). Similar aggregate costs were reported by users on the ECIGSSA forum (Supplementary file Table S2).

**Table 2:** Estimated cost of e-cigarette use among habitual users, South Africa, 2020

*Budget Line*	*Description*	*Frequency of purchase*	*Estimated cost – Price minimization options (ZAR)*	*Estimated cost – Retail option (ZAR)*	*Total annual cost – Price minimization options (ZAR)*	*Total annual cost – Retail option (ZAR)*
**Mod device**	Assumes only 1 device will be purchased. A ‘mod’ is assumed because of greater efficiency than a disposable and greater ability to customize the vaping sensory experience.	One-time cost in a year	812.19 (non-DIY device)	812.19 per mod device		
Total cost					812.19	812.19
**Battery**	3000 mAh, 15 A used as standard.	Once monthly replacement	210 per month (non-DIY device) or 2520 per year	210 per month or 2520 per year		
Total cost					2520	2520
**Refill juice**	For the retail option, we used prices reflective of national average prices for pre-mixed e-juice based on current market rates and typical consumption patterns. For the price minimizing strategy option, we assume the user mixes their own vape juice from concentrates.	Regardless of whether using pre-mixed or self-mixed liquid, refill frequency will depend on usage patterns and device characteristics. Review of e-liquid consumption rates from ECIGSSA showed a low of 140 mL per week (about 20 mL per day) to a high of 200 mL per week (about 60 mL of juice every 2nd day, or ‘pretty much’ 2 × 100 mL per week). Assuming e-cigarette users on this platform are aficionados, we took half of the weekly consumption reported on this platform (200 mL/2 = 100 mL) as average for a typical user.	The most common pack size for refill liquid concentrate was 10 mL, which yields 100 mL of vape liquid on mixing (per manufacturer instructions). Average price of 1 pack of 10 mL concentrate was 83.51 per week or 4342.5 per year	Average cost of 100 mL of nicotine-containing regular e-cigarette liquid was 274.44. At consumption rate of 100 mL per week, expenditures for e-liquid would therefore amount to 274.44 per week or 14270.88 per year		
Total cost					4342.5	14270.88
**Coils**	Heats the e-liquid to create the vapor which is inhaled by the user.	Two new coils per month assumed based on ‘medium’ vaping frequency^20^	25 per coil, 50 per month or 600 per year	78.24 per retail coil, 156.48 per month or 1877.76 per year		
Total cost					600	1877.76
**Organic cotton**	Wick material for coil building. Cotton wicks need more frequent replacement as they soil faster. ‘Sweeter’ juices may also need more frequent re-wicking.	Two packs per month	300 for 2 packs	300 for 2 packs		
Total cost					300	300
**Total annual cost**					8574.69	19780.83

ZAR: South Africa Rand (about US$ 69 to 1000 ZAR). DIY: do-it-yourself.

**Figure 1:**
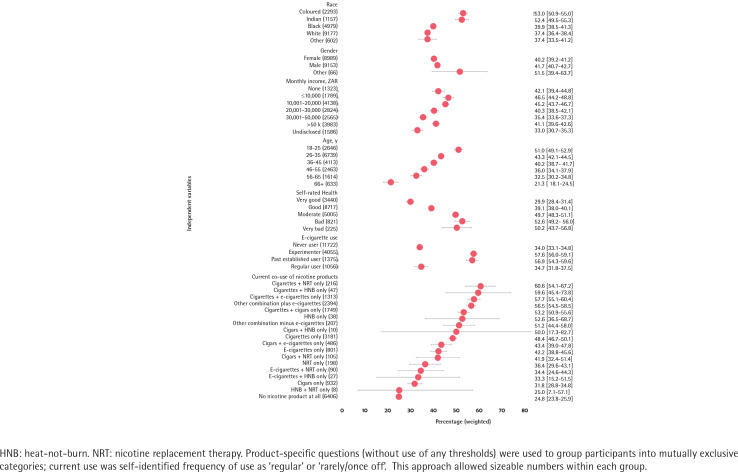
Percentage of South African adults aged ≥18 years who perceived that ‘e-cigarettes vaping and heat-not-burn devices... are too expensive to use’ 2018 (N=18208)

Among the 18208 adult participants in the Health 24 survey, 36.5% perceived e-cigarettes were ‘too expensive’, including 34.0% of e-cigarette never users, 57.6% of e-cigarette experimenters, 56.9% of past established users, and 34.7% of current established users (Figure 1).

### Potential revenue from taxation of e-cigarettes in South Africa

Our calculations show that taxing e-cigarettes at 75% of the current cigarette excise tax rate can generate annual revenue of up to ZAR 2.20 billion (95% CI: 0.96–3.44) (Table 3). If taxed at 37.5% of the current cigarette tax rate – half of the proposed rate for e-cigarettes by the government – the projected revenue amounts to ZAR 1.10 billion (95% CI: 0.48–1.72) (Table 4). Estimates were very robust under varying assumptions.

**Table 3:** Estimated and projected revenue (in ZAR) from taxation of e-cigarettes in South Africa at 75% of current cigarette excise tax rate, 2018–2021

*Frequency of e-cigarette use*	*Weighted prevalence ^a^*	*Weighted population counts from prevalence ^b^*	Weighted counts of e-cigarette users in current calendar year by assumption*	*Number of packages of 10 mL e-liquid concentrate purchased from retailers ^c^*	*2018 Annual tax on e-liquid per person ^d^*	*2018 Annual excise tax on e-liquid, aggregate ^e^*	*2018 Annual excise tax on hardware (device plus batteries), aggregate ^f^*	*Total excise tax for 2018 (e-liquid and hardware combined) ^g^*	*2021 projected excise tax based on market forecasts for consumption patterns ^h^*
**Point estimate**									
Currently every day	0.73	295081	295081	52	605.28	178606628	223047098	401653726	
Currently some days	1.98	794936	794936	39	453.96	360869147	600879650	961748796	
Stopped <6 m ago	0.8	320003	320003	26	302.64	96845708	150421251	247266959	
Stopped >6 m ago (ASM1)*	0.58	231369	231369	13	151.32	35010757	75692565	110703322	
Stopped >6 m ago (ASM2)	0.58	231369	0	0	0	0	0	0	
Stopped >6 m ago (ASM3)	0.8	320003	320003	13	151.32	48422854	104689254	153112108	
Total ASM1						671332239	1050040565	1721372804	2150387449
Total ASM2						636321482	974347999	1610669482	2012093737
Total ASM3						684744336	1079037253	1763781589	2203365700
**Lower limit**									
Currently every day	0.43	135038	135038	52	605.28	81735801	102073105	183808906	151538219
Currently some days	1.18	380902	380902	39	453.96	172914272	287917845	460832117	
Stopped <6 m ago	0.4	100970	100970	26	302.64	30557561	47462161	78019721	
Stopped >6 m ago (ASM1)*	0.31	89596	89596	13	151.32	13557667	29311408	42869074	
Stopped >6 m ago (ASM2)	0.31	89596	0	0	0	0	0	0	
Stopped >6 m ago (ASM3)	0.4	100970	100970	13	151.32	15278780	33032421	48311202	
Total for ASM1						298765300	466764519	765529819	956321438
Total for ASM2						285207633	437453111	722660745	902768181
Total for ASM3						300486414	470485533	770971947	963119897
**Upper limit**									
Currently every day	1.26	455123	455123	52	605.28	275476849	344020335	619497184	66239333
Currently some days	3.3	1208971	1208971	39	453.96	548824475	913842210	1462666685	
Stopped <6 m ago	1.57	539036	539036	26	302.64	163133855	253380342	416514197	
Stopped >6 m ago (ASM1)*	1.06	373141	373141	13	151.32	56463696	122073396	178537092	
Stopped >6 m ago (ASM2)	1.06	373141	0	0	0	0	0	0	
Stopped >6 m ago (ASM3)	1.57	539036	539036	13	151.32	81566928	176346086	257913013	
Total for ASM1						1043898876	1633316283	2677215159	3344452674
Total for ASM2						987435180	1511242887	2498678067	3121419104
Total for ASM3						1069002107	1687588973	2756591080	3443611313

* ASM: Assumption. ASM1: All of those who stopped >6 m ago use in current calendar year. ASM2: None of those who stopped >6 m ago used an e-cigarette in current calendar year. ASM3: The number who quit e-cigarette use in the latter half of the year is the same as the number who quit in the first half of the year.

a Computed from the 2018 South African Social Attitude Survey (N=2736). Data were weighted to yield nationally representative estimates of the South African population aged ≥16 years. In the 2018 SASAS dataset, unweighted number of observations for e-cigarette use were: currently every day (22); currently some days (40); stopped completely less than 6 months ago (19); stopped completely more than 6 months ago (22).

b Extrapolated to generate actual counts of individuals aged ≥16 years in South Africa based on census data. Weights available within the dataset were used for extrapolation.

c All calculations were performed for 31 December of the calendar year and apply for the whole year. We assume that in the presence of excise tax, most e-cigarette users will mix their own liquid as a tax minimizing strategy. A 10 mL package of e-juice concentrate yields 100 mL of ready-to-use vape liquid. Based on assumed rates of e-liquid consumption (100 mL of liquid per week), we estimate that daily users will consume 1 pack of 10 mL concentrate each week, or 52 packs for the entire year. Those who only use e-cigarettes ‘some days’ are assumed to consume e-liquid at 75% of the rate of daily users (i.e. they use all year round, but at reduced intensity). Those who stopped completely <6 m ago are assumed to have used e-cigarettes for half of the year; their rate of consumption of e-cigarettes during the year is therefore assumed to be 50% that of daily users (i.e. they used half of the year, but at the same intensity as daily users when they were actively using). Those who stopped completely >6 m ago are assumed to have used e-cigarettes for a quarter of the year; their rate of e-liquid consumption is therefore assumed to be 25% that of daily users (i.e. they used a quarter of the year, but at the same intensity as daily users when they were actively using).

d Obtained by multiplying ZAR 11.64 by the number of expected packs consumed during the year; ZAR 11.64 is derived as 75% of the cigarette excise tax of ZAR 15.52 per cigarette pack during 2018. The threshold of 75% was the proposed tax rate by the Minister of Finance for novel tobacco products.

e Calculated by multiplying the total annual tax per person by the number of persons in that bracket of e-cigarette users.

f ‘Hardware’ includes both the device itself and the battery. From our online vendor data, average market prices, inclusive of VAT, were ZAR 812.19 for ‘mod’ devices (averaged across different types), and ZAR 210 for batteries. We assume 1 device per year and monthly battery replacements.

g Projections for excise tax were based on pre-VAT prices; at 15% VAT, this yields ZAR 706.25 for a device and ZAR 182.61 for batteries. For current users (daily and non-daily users), total hardware cost applied for excise tax projection was ZAR 706.25 + (182.61×12) = 2897.56, assuming use of e-cigarettes all 12 months of the year. For those assumed to have used e-cigarettes for half of the year (quit <6 months ago), this was ZAR 706.25 + (182.61×6) = 1801.90. For those assumed to have used e-cigarettes for a quarter of the year (quit >6 months ago), this was ZAR 706.25 + (182.61×12) = 1254.08. The excise tax levied on these amounts was assumed at a rate of 30% which is 75% of the cigarette excise tax rate of 40%. The tax paid per individual for e-cigarette hardware per year was therefore ZAR 756 for current users, ZAR 470 for those assumed to have used for 6 months, and ZAR 327 for those assumed to have used for 3 months only. Projected tax from hardware was generated by multiplying these amounts by the total number of e-cigarette users in each bracket for the year.

h Based on projected growth in e-cigarette consumption by 24.9% during 2018–2021 as forecasted by Euromonitor International.

**Table 4:** Estimated and projected revenue (in ZAR) from taxation of e-cigarettes in South Africa at 37.5% of current cigarette excise tax rate, 2018–2021

*Frequency of e-cigarette use*	*Weighted prevalence ^a^*	*Weighted population counts from prevalence ^b^*	Weighted counts of e-cigarette users in current calendar year by assumption*	*Number of packages of 10 mL e-liquid concentrate purchased from retailers ^c^*	*2018 Annual tax on e-liquid per person ^d^*	*2018 Annual excise tax on e-liquid, aggregate ^e^*	*2018 Annual excise tax on hardware (device plus batteries), aggregate ^f^*	*Total excise tax for 2018 (e-liquid and hardware combined) ^g^*	*2021 projected tax based on market forecasts for consumption patterns ^h^*
**Point estimate**									
Currently every day	0.73	295081	295081	52	302.64	89303314	111523549	200826863	
Currently some days	1.98	794936	794936	39	226.98	180434573	300439825	480874398	
Stopped <6 m ago	0.8	320003	320003	26	151.32	48422854	75210626	123633480	
Stopped >6 m ago (ASM1)*	0.58	231369	231369	13	75.66	17505379	37846283	55351661	
Stopped >6 m ago (ASM2)	0.58	231369	0	0	0	0	0	0	
Stopped >6 m ago (ASM3)	0.8	320003	320003	13	75.66	24211427	52344627	76556054	
Total ASM1						335666120	525020282	860686402	1075193725
Total ASM2						318160741	487174000	805334741	1006046869
Total ASM3						342372168	539518627	881890795	1101682850
**Lower limit**									
Currently every day	0.43	135038	135038	52	302.64	40867900	51036553	91904453	75769109
Currently some days	1.18	380902	380902	39	226.98	86457136	143958923	230416059	
Stopped <6 m ago	0.4	100970	100970	26	151.32	15278780	23731080	39009861	
Stopped >6 m ago (ASM1)*	0.31	89596	89596	13	75.66	6778833	14655704	21434537	
Stopped >6 m ago (ASM2)	0.31	89596	0	0	0	0	0	0	
Stopped >6 m ago (ASM3)	0.4	100970	100970	13	75.66	7639390	16516211	24155601	
Total ASM1						149382650	233382260	382764910	478160719
Total ASM2						142603817	218726556	361330372	451384091
Total ASM3						150243207	235242766	385485973	481559949
**Upper limit**									
Currently every day	1.26	455123	455123	52	302.64	137738425	172010167	309748592	33119666
Currently some days	3.3	1208971	1208971	39	226.98	274412238	456921105	731333343	
Stopped <6 m ago	1.57	539036	539036	26	151.32	81566928	126690171	208257099	
Stopped >6 m ago (ASM1)*	1.06	373141	373141	13	75.66	28231848	61036698	89268546	
Stopped >6 m ago (ASM2)	1.06	373141	0	0	0	0	0	0	
Stopped >6 m ago (ASM3)	1.57	539036	539036	13	75.66	40783464	88173043	128956507	
Total ASM1						521949438	816658141	1338607579	1672226337
Total ASM2						493717590	755621444	1249339033	1560709552
Total ASM3						534501054	843794487	1378295540	1721805657

* ASM: Assumption. ASM1: All of those who stopped >6 m ago use in current calendar year. ASM2: None of those who stopped >6 m ago used an e-cigarette in current calendar year. ASM3: The number who quit e-cigarette use in the latter half of the year is the same as the number who quit in the first half of the year.

a Computed from the 2018 South African Social Attitude Survey (N=2736). Data were weighted to yield nationally representative estimates of the South African population aged ≥16 years. In the 2018 SASAS dataset unweighted number of observations for e-cigarette use were: currently every day (22); currently some days (40); stopped completely less than 6 months ago (19); stopped completely more than 6 months ago (22).

b Extrapolated to generate actual counts of individuals aged ≥16 years in South Africa based on census data. Weights available within the dataset were used for extrapolation.

c All calculations are performed for 31 December of the calendar year and apply for the whole year. We assume that in the presence of excise tax most e-cigarette users will mix their own liquid as a tax minimizing strategy. A 10 mL package of e-juice concentrate yields 100 mL of ready-to-use vape liquid. Based on assumed rates of e-liquid consumption (100 mL of liquid per week) we estimate that daily users will consume 1 pack of 10 mL concentrate each week or 52 packs for the entire year. Those who only use e-cigarettes ‘some days’ are assumed to consume e-liquid at 75% of the rate of daily users (i.e. they use all year round but at reduced intensity). Those who stopped completely <6 m ago are assumed to have used e-cigarettes for half of the year; their rate of consumption of e-cigarettes during the year is therefore assumed to be 50% that of daily users (i.e. they used half of the year but at the same intensity as daily users when they were actively using). Those who stopped completely >6 m ago are assumed to have used e-cigarettes for a quarter of the year; their rate of e-liquid consumption is therefore assumed to be 25% that of daily users (i.e. they used a quarter of the year but at the same intensity as daily users when they were actively using).

d Obtained by multiplying ZAR 5.82 by the number of expected packs consumed during the year; ZAR 5.82 is derived as 37.5% of the cigarette tax of ZAR 15.52 per cigarette pack during 2018. The threshold of 37.5% was applied as this was half of the 75% proposed tax rate by the Minister of Finance for novel tobacco products.

e Calculated by multiplying the total annual tax per person by the number of persons in that bracket of e-cigarette users.

f ‘Hardware’ includes both the device itself and the battery. From our online vendor data average market prices inclusive of VAT were ZAR 812.19 for ‘mod’ devices (averaged across different types) and ZAR 210 for batteries. We assume 1 device per year and monthly battery replacements.

g Projections for excise tax were based on pre-VAT prices; at 15% VAT this yields ZAR 706.25 for a device and ZAR 182.61 for batteries. For current users (daily and non-daily users) total hardware cost applied for excise tax projection was ZAR 706.25 + (182.61×12) = 2897.56 assuming use of e-cigarettes all 12 months of the year. For those assumed to have used e-cigarettes for half of the year (quit <6 months ago) this was ZAR 706.25 + (182.61× 6) = 1801.90. For those assumed to have used e-cigarettes for a quarter of the year (quit > 6 months ago) this was ZAR 706.25 + (182.61×12) = 1254.08. The excise tax levied on these amounts was assumed at a rate of 15% which is 37.5% of the cigarette excise tax rate of 40%. The tax paid per individual for e-cigarette hardware per year was therefore ZAR 378 for current users ZAR 235 for those assumed to have used for 6 months and ZAR 164 for those assumed to have used for 3 months only. Projected tax from hardware was generated by multiplying these amounts above by the total number of e-cigarette users in each bracket for the year.

h Based on projected growth in e-cigarette consumption by 24.9% during 2018–2021 as forecasted by Euromonitor International.

Of the projected e-cigarette excise tax at 75% of the cigarette tax rate, the portion attributable to hardware (device and batteries) was 61% (ZAR 1.35 billion), while the portion attributable to e-liquid was 39% (ZAR 0.86 billion).

Our sensitivity analyses showed that our expected excise tax revenue from cigarettes aligned with official estimates from the South African Treasury. From our three scenario analyses under varying assumptions, the median expected excise tax in 2018 was ZAR 11.57 billion (scenario 1: 11.84 billion; scenario 2: 11.45 billion; scenario 3: 11.57 billion, Table 5), compared to the observed value of ZAR 12.5 billion in 2018–2019.

**Table 5:** Sensitivity analysis showing expected revenue (in ZAR) from taxation of manufactured cigarettes in South Africa at 40% excise tax rate 2018

*Frequency of smoking manufactured cigarettes*	*Weighted prevalence ^a^*	*Weighted population counts from prevalence ^b^*	*Mean cigarettes smoked per day (selfreported) ^c^*	*Mean cost per cigarette pack (gross self-reported) ^d^*	*Pre-VAT price per cigarette pack (excluding 15% value added tax) ^e^*	*Portion of year smoked (assumed)*	*Total manufactured cigarettes smoked per year/person (single sticks) ^f^*	*Total manufactured cigarettes smoked per year/person (packs of 20 sticks) ^g^*	*Total expenditures on manufactured cigarettes per year for all smokers at net price (exclusive of VAT) ^h^*	*Expected excise tax generated assuming no illicit trade ^i^*	*Expected excise tax accounting for illicit trade (33.21%) ^j^*
Currently every day	15.4	6196978	12.02	30.94	26.90	100 (365 d)	4387	219	36569117162	14627646865	
Currently some days	5.0	2013620	7.34	30.94	26.90	75 (274 d)	2010	100	5444415656	2177766262	
Stopped <6 m ago	0.7	290850	12.02	30.94	26.90	50 (183 d)	2193	110	858170525	343268210	
Stopped >6 m ago ASM1*	2.5	991440	12.02	30.94	26.90	25 (91 d)	1097	55	1462651857	585060743	
Stopped >6 m ago ASM2	2.5	991440	0	0	0	0 (0 d)	0	0	-	-	
Stopped >6 m ago ASM3	0.7	290850	12.02	30.94	26.90	25 (91 d)	1097	55	429085262	171634105	
Total ASM1^j^										17733742080	11844366335
Total ASM2^j^										17148681337	11453604265
Total ASM3^j^										17320315442	11568238684

* ASM: Assumption. ASM1: All of those who stopped >6 m ago smoked a manufactured cigarette in current calendar year. ASM2: None of those who stopped >6 m ago smoked a manufactured cigarette in current calendar year. ASM3: The number who quit manufactured cigarette smoking in the latter half of the year is the same as the number who quit in the first half of the year. All calculations are performed for 31 December of the calendar year.

a Computed from the 2018 South African Social Attitude Survey (N=2736). Data were weighted to yield nationally representative estimates of the South African population aged ≥16 years.

b Extrapolated to generate actual counts of individuals aged ≥16 years in South Africa based on census data; internal weights were used for extrapolation.

c Computed from the 2018 South African Social Attitude Survey. The survey question was ‘On the days that you smoke, on average, how many manufactured cigarettes (excluding hand rolled cigarettes) do you smoke per day?’. For persons who had quit data were not available on their intensity of smoking when they smoked; we assumed they smoked at the intensity of daily smokers during the period they smoked in the year.

d Computed from the 2018 South African Social Attitude Survey. The survey question was: ‘How much did you pay for your last cigarette purchase per pack?’. Cost estimates are in South African Rand (ZAR). The ZAR 30.94 estimate was from all participants who reported buying cigarettes in a pack and this mean estimate was applied to all smokers. For those who purchased cigarettes in single sticks the mean cost of ZAR 30.94 was applied to their cigarette-pack equivalents, based on total consumption in the entire year.

e Derived by removing the 15% value added tax from gross price of ZAR 30.94.

f Derived by multiplying mean number of cigarettes smoked per day by assumed number of days smoked in the entire year.

g Derived by dividing total number of individual cigarettes smoked in the year by 20 (standard number of individual cigarettes in a pack).

h Projected expenditure was generated by multiplying the total number of manufactured cigarette smokers in each bracket × estimated number of cigarette packs smoked in the year × mean cost of a cigarette pack (ZAR 30.94).

i Derived by multiplying total cigarette expenditures by 0.4, the cigarette excise tax rate (40%).

j The extent of illicit trade was estimated in 2018 SASAS with the question: ‘Overall, how many of the cigarettes that you have smoked could possibly be counterfeit or illegal (tax not paid/smuggled)?’. Response options were ‘none’, ‘a little’, ‘about half’, ‘most’, ‘all’, and ‘can't say’. The unit of analysis was the individual respondents (percentage of individuals). We used the reported percentages as a crude marker for number of illicit products (percentage of cigarettes). Using these responses we estimated the magnitude of illicit trade as 33.21% (sum of the following percentage points: percentage answering ‘none’ [32.53%] × 0, percentage answering ‘a little’ [28.63%] × 0.25, percentage answering ‘about half’ [18.77%] × 0.5, percentage answering ‘most’ [13.63%] × 0.75, and percentage answering ‘all’ [6.44%] × 1.0).

## DISCUSSION

Findings from our study do not support industry’s narrative in e-cigarette marketing asserting that e-cigarette use is a cheaper alternative to cigarette smoking^8^. One industry study, which compared daily consumption of e-cigarettes and cigarettes, claimed that e-cigarette use is 7% less expensive than smoking for the average smoker (ZAR 8307 and 9724 for the average e-cigarette user and cigarette smoker, respectively)^8^. Conversely, we found that the average annual cost associated with daily smoking of manufactured cigarettes was ZAR 6693; in contrast, e-cigarette-related costs ranged from ZAR 8574.69 per year (with DIYs) to ZAR 19780.83 (using retail products exclusively).

Implementing excise taxes on e-cigarettes may generate revenue for the public good in general, including for comprehensive tobacco control/prevention efforts. In the United States, recommended funding for tobacco control in the 50 states and D.C. for fiscal year 2019 by the Centers for Disease Control and Prevention was US$66869056 (ZAR 0.97 billion)^18^. The results from our study show that taxing e-cigarettes at the rate proposed by the South African Minister of Finance (75% of the current cigarette excise tax rate) can generate annual revenue of up to ZAR 2.20 billion, or if taxed at half of the official proposed rate (37.5%), annual revenues of up to ZAR 1.10 billion.

E-cigarette tax structure could easily become unwieldy because of the sheer number of variables to consider, including but not limited to the device, liquid type, other consumables, frequency of use, and nicotine concentration; this complexity may explain the wide variation in tax structure among countries that have taxed e-cigarettes^19^. While the National Treasury in South Africa has indicated intentions of taxing e-cigarettes^20^, there is no clear blueprint of how this will be implemented. Kenya is the only African country that currently taxes e-cigarettes^19^. As South Africa considers e-cigarette taxation^15^, it will be important to prevent loopholes for the industry to exploit, such as mislabeling products or shifting the dominant products sold to avoid taxes^20,21^. We recommend that the following be considered in relation to taxes for e-cigarettes in South Africa: 1) taxes should apply to any liquid designed to be used with an e-cigarette device, or any liquid sold by an e-cigarette vendor, online or in a brick-and-mortar store, regardless of the liquid’s ingredients, additives, nicotine content or other characteristics (e.g. CBD liquid being sold in vape shops for use with e-cigarettes should be classified as e-liquid for the purpose of taxation; and 2) taxes should be based on a characteristic for which there would be strong disincentive for e-cigarette manufacturers to alter product label, the most ideal metric would be package volume. Findings from our study showed that e-liquid prices were independently associated with the volume, but not the concentration of the e-liquid. In other words, mislabeling the package volume to avoid higher taxes may hurt the profit margins of e-cigarette manufacturers much more than mislabeling the nicotine content of e-liquids being sold. Use of package volume is desirable for the additional reason that it is more easily verifiable in real-time and onsite by public health authorities during compliance checks, compared to nicotine content which may require further laboratory analyses.

There are several tradeoffs involved in a tax on e-cigarettes, the quantification of which is beyond the scope of this study. The net impact on public health depends on: the number of adult users versus the number of potential youth users; the health gains to adults who use e-cigarettes to quit smoking versus the health losses of youth who use e-cigarettes; and the relative responsiveness of adult and youth use to a tax-induced increase in the price of e-cigarettes. Further research on these policy issues is needed.

### Limitations

This study has some limitations. First, our analysis did not incorporate an estimate of the price-elasticity of consumer demand for e-cigarettes specific to South Africa from empirical data. We also assumed that illicit trade is negligible for e-cigarettes. Second, the data from SASAS were self-reported and may be subject to misclassification. Finally, our estimates of potential revenue from e-cigarette taxation are very conservative. For example, we assume that each user purchases only 1 device per year, which ignores the fact that some users may have multiple devices concurrently or buy multiple replacement devices in a single year.

## CONCLUSIONS

Contrary to claims made by e-cigarette manufacturers, a comparison of reasonably ‘exchangeable’ groups of cigarette smokers and e-cigarette users found annual costs from e-cigarette use much higher compared to cigarette smoking. We recommend levying an e-cigarette excise tax. The government’s proposed tax rate may help generate revenue and reduce youth e-cigarette access, while allowing adult smokers wishing to switch exclusively to e-cigarettes reduce their tobacco-related harm.
