# Supplementary material for: Potential revenue from taxing e-cigarettes and comparison of annual costs of daily e-cigarette use versus daily cigarette smoking among South African adults
Source: Tob Induc Dis. 2021 Jan 29;19:07. doi: 10.18332/tid/131861 (PMC7842579; doi:10.18332/tid/131861)
Supplement: Supplementary file 1 [file TID-19-07-s1.pdf]

Table S1. Overview of data sources used in the study

Name	South African Social Attitudes Survey (SASAS)	Health 24 Survey	Online vendor data	Ecig Vape Forum South Africa (ECIGSSA)
**Data collected by**	Human Science Research Council	News 24	Author IA	Author IA
**Target population**	South African adults aged 16+ years	South African adults aged 18+ years	Individual e-cigarette products sold online by vendors in South Africa	South Africa's largest e-cigarette forum, with >8,000 members
**Type of data**	Structured, quantitative	Structured, quantitative	Structured, quantitative	Unstructured, free text
**Mode of data collection**	Household survey, in-person	Web-based survey	Web scraping/harvesting of online data from retail websites	Online forum discussion
**Year**	2018	2018	2020	2019/2020
**Sample size**	2736	18,208	2,661 refill liquids and 171 hardware	20 responses analyzed
**Unit of analyses**	People	People	Products	People
**Use in paper**	To generate the following estimates: (1) Prevalence (%) and weighted population counts (millions) of e-cigarette and manufactured cigarette use in South Africa (2) To generate estimates of prevalence of use of illicit cigarettes within sensitivity analyses (3) To generate estimates of mean cigarettes smoked per day (4) To generate estimates of cost of cigarettes by individual sticks or by packs using self-reported information	To estimate the percentage of South African adults who perceived that "e-cigarettes, vaping, and heat-not-burn devices… Are too expensive to use"	(1) To estimate costs of e-cigarette retail products on the South African market (2) To assess the variations in product characteristics, including price, flavors, nicotine concentration, and other packaging elements	To assess patterns of e-cigarette use among e-cigarette users (2) To get estimates of frequency of e-cigarette use and average amounts of e-liquid consumed weekly among habitual e-cigarette users (3) To assess price minimization strategies among habitual e-cigarette users.

**Survey questions analyzed**	"On the days that you smoke, on average, how many manufactured cigarettes, (excluding hand rolled cigarettes) do you smoke per day?" (2) "Do you use or have you used any of the following tobacco products?" - "Manufactured Cigarettes", "Electronic cigarettes (vapour cig)" (3) "How much did you pay for your last cigarette purchase, per pack?" (4) "Overall, how many of the cigarettes that you have smoked could possibly be counterfeit or illegal (tax not paid/smuggled)?"	"To what extent do you agree or disagree with each of these statements about smokeless products, such as e-cigarettes, vaping and heat-not-burn devices?- Are too expensive to use"	The online vendor data were extracted by using the Extract, Transform, and Load (ETL) methodology. An Excel spread sheet was first created, containing the web links of each store/vendor. The Extraction and Transformation process was implemented with Python Scripts (BeautifulSoup, urllib) whereas the Loading was done manually. Overall, this was an iterative process to extract data for 231 websites. The script was altered each time for each different web page to extract the data successfully. Data extraction success rate was between 80-85%.	Author IA registered on the forum on April 9, 2019 and posted the following question: “Curious how much variation in total MONTHLY cost from vaping is within the vaping community and how this differs between newbies vs versus the more experienced vapers. Please, can you tell me your average monthly cost and how long you have been vaping?”

Table S2. Responses by members of ECIGSSA ("Ecig Vape Forum South Africa") to the question "Curious how much variation in total MONTHLY cost from vaping is within the vaping community and how this differs between newbies vs the more experienced vapers. Please, can you tell me your average monthly cost and how long you have been vaping?"

#1	Very interesting question and the answers will vary from a few hundreds to a few thousands depending on:<br>1. whether you buy commercial juice, or you DIY [Do it yourself] yours<br>2. And most importantly whether you know and found what you want in terms of vape gear or you are still hunting for the perfect setup.<br><br>The actual question seen that you are new to vaping is are you still on the stinkies [regular cigarettes] or are you exclusively vaping.<br>My advice is, go through the different threads in the newbies section and don't hesitate to ask for advice. We have a very helpful community and veterans with enough expertise that can give you guidance.
#2	Avg monthly cost:<br>Juice (pretty much 2 x 100ml a week, plus the odd new one to try) - R2500 to R3000<br>Cotton x 2 packs - R300<br>Coils x 12 - R300 (these are handmade so are cheaper than retail ,which would be around R1200)<br>Batteries - R1200 every 6 months, so R200 per month<br><br>Mods and atties - fairly cheap at first but sky's the limit once it becomes a hobby and more than just a nicotine replacement mechanism.
#3	Like they said... the most costly part for me was finding that sweet spot (vape setup and favorite juice). It could cost you anything from R1000 to 100k in 12 months... hi end, entry level, MTL [Mouth to Lung], DL [Direct Lung], small, big, … 3mg 6mg,nic salts, fruity, cream… the list goes on forever !!!!)<br>BUT<br>Once that is done, ave monthly cost is about R3000, then you do not hold back., but sure you can get away with R2000 if you budget.
#4	My monthly Vape Budget is...<br><br>No I can't... what I can say is it's less than what it would cost me ICU [intensive care unit] if I was still smoking!

#5	Agree with [#1] , if you are looking at vaping simply as a smoking cessation mechanism, it can be fairly cheap and in most cases cheaper that cigarettes (Pod system, some coils, some nic salts or high freebase nicotine juice). Vaping gets "expensive" when it becomes a hobby and you start trying different mods, atomizers, juices, premium coils, etc. I personally go through about 60mls of juice every 2nd day, so it is getting quite expensive for me personally. That is why I recently made the choice to start DIY'ing for myself. I will still buy commercial juice, but just less frequently. Hardware is not something I upgrade on regularly and mostly only out of necessity. Also, if I had something like this forum when I began, I could have saved myself a lot of money in the beginning, because I bought a ton of nonsense that was "recommended" to me by sales-happy vapeshops, instead of what I needed at that stage.
#6	Don’t believe everything you read on the internet.<br>Vaping can be pretty cheap. You first month or two can be a bit expensive to settle on what you like. But if you buy from the classifieds it’s not bad. For about 2-3k you can buy 2-3 mods, batteries, chargers, cotton, coils, and tools. If you buy on classifieds you can sell it at more or less the same price again if you [are] not happy with it. My ciggies [cigarettes] cost was about 2k a month, so this mod and stuff buying the first month or two did not bother me much.<br><br>Now that I’m settled my monthly expenditure is less than R500. That’s only for juice. I vape about 20ml a day, so 1 Red pill oneshot [concentrates], one Cool blood oneshot cost R220 and gives +-520ml juice. Plus PG, VG and some nic and I mix another 100ml of my own recipe. All together less than R500.<br><br>Cotton and wire for making my own coils I don’t include, because a packets of Cotton bacon and roll of ni80 wire will cost +-R250 and can last 6-12months. Batteries also don’t need to replaced that often so I don’t regard that as an cost.<br><br>I only keep what I use, if I want something else, I sell whatever I’m then not going to use<br>I’m vaping about 6mnths
#7	My monthly vape bill is around 10 times the amount my wife thinks it is costing me
#8	I started vaping a year ago. Like others have said, I spent quite a bit at first trying to find the perfect mod and tank setup for me. I DIY juice and coils, and now pretty much only spend around R500 every second month or so.<br><br>That being said, I don't vape a lot anymore, but it helps to keep me from smoking especially when I've had a beer or 9.

#9	I vape for around R200 a month now. That is only possible with DIY for coils and juice. It cost me a lot to reach this point, buying all the devices, batteries, charger, tools, concentrates, scale, bottles, etc. But once you've done the heavy lifting early on, it gets crazy cheap.<br><br>It's really just a case of restocking concentrates that get used up, bases like nic, VG and PG, and then consumables like cotton and wire. Every two years or so I will replace batts, but I can afford to wait for specials on those. Once a year or so, I'll get a new dripper. And then replacing mods that fail, which happens probably every two years or thereabouts. And that's about it.
#10	My monthly costs are about R50. I spent 25k to get there though.<br><br>With DIY, Nicsalts, and rebuildable MTL tank<br><br>I don't even consider vaping an expense anymore.<br><br>R1000's went into the learning process
#11	Having bought and sold mods and tanks, the wife and I are with the following;<br>2 x dual battery mods + Dual coil tanks<br>1 x single battery squonker<br>2 x pod devices<br>6 x batteries<br>2 x extra sets of crafted coils<br>5 x bags and tubs of cotton<br><br>I don't see me spending ANY money on anything besides juice in a year or so; R300 p/m or so on juice.
#12	In 2017 it averaged around 8k a month for me. 2018 around 3k.
#13	Spent way too much in the beginning, and the same with DIY as well.<br><br>Consolidating now and soon it will only be concentrates for DIY maintenance. Already down from about +- 1000 to 2000 a month spread over the 18 months or so since I started to about 400 to 800 pm [per month] for supplies, but still have months where this climbs when those just want to need items still come up, but it’s getting less frequent.<br><br>Slow and easy is the way to do it, the Big Bang theory costs too much!
#14	Budget! I don’t need no stinking Budget!

#15	Seems like this question should have been "how long is a piece of string"<br><br>I think a better question would be "what do you spend on vaping as a percentage of your salary" although a bit too personal really.<br><br>Because someone earning 50k+ would be less likely to bother with DIY - increasing their costs while still buying products that cost more simply because more expensive is usually better right?. Whereas someone on the 3-5K mark is going to try their absolute best to save a buck.<br><br>Then there's the issue of this forum. Most people here are not your average vaper. This forum, imo, represents the hobbyists and extremists with regards to vaping.<br><br>How often do we see a smok X8 or ego AIO handcheck? Yet these devices still fly out of vape stores.<br><br>Consumers are usually broken into tiers and then evaluated or marketed to accordingly. No point advertising your Merc to a chap who won't be able to pay it off over his lifetime even if he had no other expenses.<br><br>You get smokers who vape - even guys deeply into the vape scene - our local juice makers some of them still smoke.<br><br>I can appreciate wanting to get a rough idea, but I don't believe it's possible to draw any kind of conclusion from the results.
#16	*[Response to #15] Agree, but not with the salary thing. I often see the same argument regarding HE gear. I have no problem to go spend 3k on a fishing reel. But I will never spend that on a vape mod, probably not even 1k, its not important to me. Then I know people that really has little money and can barely pay their rent that has much more expensive fishing gear that me. But that guy dreamed of it, and saved every sent he could tlll he could buy his dream.*<br>*My best friend is a elec engineer and his wife a surgeon. He drives a Honda accord and she a Fiesta, because they just can’t be bothered about what car they drive*
#16	*[Response to #15] Simply put, for me personally, I've spent more on juice than I have on gear*
#18	*[Response to #15] Once again, throwing another spanner into the works everyone earns different amounts, no two personalities are identical. One man's trash, all that jazz. I agree with you. 100%*

#19	Costs all depend on your intentions and approach.<br>If you just want a replacement for a nicotine craving then a cheap pod system and nic salts could work out very cheap in the long run .... less than R200 a month.<br><br>If you get caught by the flavor and cloud bug then larger more expensive devices will be sought which could lead to thousands of rands per month.<br><br>This cost could be brought down drastically if you are willing to learn and start making your own coils and ejuice. We roughly talking about R500 per month.<br><br>The hardest thing will be finding the perfect setup for your style of vaping. Of course a cheaper back-up setup will be a good idea but once you have them all should be smooth sailing, regarding your bank account, from that point on.<br><br>Vaping cost me alot but it is because that was my decision to take the habit beyond what was really necessary.<br><br>A budget approach could be to try the different devices of other vapers till you get that smile and just go for that one instead of buying a plethora of your own in the hope to pin down the ONE.<br><br>The danger arises when vaping become a hobby and your device needs to become a symbol of desire.
#20	I'm gonna get a lot of flack for this, but if you want to save money stay away from this forum!<br>I've seen so many devices and read so many reviews that I want ALLL of them. I could probably spend R50k on all the devices I want right now and the next day I'd see something new that I'll still want.

. © 2021 Agaku I. et al.
